# A Less Common but Lethal Encounter: Mixed Autoimmune Hemolytic Anemia Unmasking Angioimmunoblastic T-cell Lymphoma

**DOI:** 10.7759/cureus.85257

**Published:** 2025-06-02

**Authors:** Saloni D Talreja, Naman Lodha, Naresh K Midha, Siddharth Mittal, Meenakshi Rao

**Affiliations:** 1 Medicine, All India Institute of Medical Sciences, Jodhpur, Jodhpur, IND; 2 Transfusion Medicine, All India Institute of Medical Sciences, Jodhpur, Jodhpur, IND; 3 Pathology, All India Institute of Medical Sciences, Jodhpur, Jodhpur, IND

**Keywords:** autoimmune hemolytic anemia (aiha), coomb's positive hemolytic anemia, fatal outcome, lymph nodes pathology, t-cell lymphoma

## Abstract

Autoimmune hemolytic anemia (AIHA) is a spectrum of acquired hemolytic disorders caused by autoantibodies targeting red blood cells, leading to their destruction and anemia. Mixed AIHA, characterized by both warm and cold autoantibodies, is a rare and complex condition, often presenting diagnostic challenges. Angioimmunoblastic T-cell lymphoma (AITL), a subtype of peripheral T-cell lymphoma, is also uncommon and can be associated with autoimmune cytopenias. We present a case of a 48-year-old male patient diagnosed with mixed AIHA and AITL after presenting with generalized weakness, jaundice, and bicytopenia. Diagnosis was confirmed through positive Coombs tests for warm and cold antibodies, lymph node biopsy, and imaging. Despite initial response to glucocorticoids, the patient experienced complications following chemotherapy initiation, leading to a fatal outcome. This case highlights the importance of early recognition of mixed AIHA and AITL and the complexities involved in their management.

## Introduction

Autoimmune hemolytic anemia (AIHA) encompasses a spectrum of acquired hemolytic disorders characterized by the development of autoantibodies targeting antigens present on the surface of the patient's own red blood cells [1], leading to the destruction of red blood cells, resulting in anemia and associated clinical manifestations. It is classified into distinct subtypes based on the temperature at which autoantibodies react with red blood cells, namely warm antibody AIHA, cold antibody AIHA and mixed AIHA. Warm antibody AIHA is characterized by autoantibodies belonging to the IgG class reacting optimally at temperatures ≥37°C and do not require complement activation for their activity. Moreover, warm antibody AIHA typically does not induce agglutination of red blood cells in vitro. In contrast, cold antibody AIHA is characterized by autoantibodies primarily of the IgM class. These antibodies react optimally at temperatures <37°C and necessitate complement activation for their hemolytic activity. A distinctive feature of cold antibody AIHA is the spontaneous agglutination of red blood cells in vitro, contributing to the clinical manifestations observed in affected individuals. In mixed AIHA, there is presence of both cold and warm autoantibodies [1]. It represents a rare and intricate diagnostic challenge, compounded by the scarcity of effective therapeutic interventions and can manifest either as an idiopathic condition or as a secondary phenomenon, often linked with systemic lupus erythematosus (SLE) and lymphoma [2].

Angioimmunoblastic T-cell lymphoma (AITL) is a distinct subtype of peripheral T-cell lymphoma (PTCL) characterized by unique clinicopathologic and genetic features. AITL accounts for a small proportion, roughly 1% to 2%, of all non-Hodgkin lymphomas. However, within the category of PTCLs, it emerges as the second most common subtype, representing approximately 15% to 20% of all cases of PTCL [3].

AITL is characterized by intense immune and inflammatory reactions. It has often been associated with autoimmune hemolytic anemia and also shows the presence of cold agglutinins, but there are very few case reports describing its association with mixed autoimmune hemolytic anemia per se. We herein report a case where the patient presented with mixed AIHA and was diagnosed to have AITL.

## Case presentation

A 48-year-old male patient, a reformed beedi smoker and a farmer by occupation, presented with a five-year history of generalized weakness, shortness of breath, increased since seven days before presentation associated with orthopnea. He had low-grade fever for two months with evening rise associated with chills occurring every one to two days, cough with expectoration for two months, loss of appetite for three months with loss of weight of around 10 kg over three months. He also gave history of yellowish discoloration of eyes since one month and swelling of bilateral lower limbs for 15 days. On presentation at the emergency department, the patient had tachycardia and tachypnoea with an oxygen saturation of 88% on room air. On examination, pallor, icterus, clubbing, generalized lymphadenopathy and bilateral pitting pedal edema were present. An abdomen examination revealed hepatosplenomegaly and on auscultation, bilateral crepitation and wheeze were present. On investigation, the patient had bicytopenia anemia and thrombocytopenia, leukocytosis, hyperbilirubinemia, elevated serum protein and albumin/globulin reversal (Table 1). Blood film morphology was showed marked anisocytosis and agglutinated red blood cells (RBCs), and serum lactate dehydrogenase was elevated. Direct Coombs test was positive for IgG, C3d and IgM, hence suggestive of mixed autoimmune hemolytic anemia. Ultrasound whole abdomen was suggestive of liver parenchymal disease with hepatosplenomegaly (liver, 15.9 cm, spleen, 15.5 cm), splenic infarcts and mild ascites. Contrast-enhanced CT chest and abdomen showed discrete non-necrotic bilateral axillary, mediastinal, mesenteric, retroperitoneal and inguinal lymphadenopathy generalized lymphadenopathy and hepatosplenomegaly with multiple peripheral splenic infarcts with a few centrilobular micro-nodules bilateral lung fields with mild right-sided pleural effusion.

**Table 1:** Laboratory Investigations ANA: anti-nuclear antibody; ASMA: anti-smooth muscle antibody; SAT: standard agglutination test; IgG: immunoglobulin G; ABG: arterial blood gas; C3 and C4: complement 3 and 4.

Investigation	Result				Reference Range
	April 10, 2024	April 12, 2024	April 21, 2024	May 2, 2024	
Hemoglobin (Hb) (g/dL)	5.1	6.2	7.9	4.8	13–17 g/dl
Total Leukocyte Count (WBC) (/cu mm)	14,500	10.7	7.12	17.5	4,000–11,000 /cu mm
Platelet Count (/µL)	19,000	23,000	37,000	24,000	150,000–450,000 /µL
Total Bilirubin (mg/dl)	3	3.57	2.57	15.6	0.1–1.2 mg/dl
Direct Bilirubin (mg/dl)	0.58	1.9	0.82	5.2	0.1–0.39 mg/dl
Total Protein (g/dl)	9.3	7.93	7.5	8	6.5–7.8 g/dl
Albumin (g/dl)	2.2	2.23	2.8	2.5	3.5–5.2 g/dl
Globulin (g/dl)	7.1	5.7	4.7	5.5	2–3.5 g/dl
Low-Density Lipoprotein (LDH) (U/L)	744	688	330	702	< 248 U/L
Urea (mg/dl)	39	58	29	47	17–43 mg/dl
Creatinine (mg/dl)	0.8	0.78	0.57	0.8	0.6–1.1 mg/dl
Direct Coombs Test	Positive (IgG, C3d, IgM)				
24-Hour Urinary Protein	1.4 g/24h				<150 mg/24h
ANA	Negative				
C3	39 mg/dl				90–180 mg/dl
C4	<6 mg/dl				10–40 mg/dl
Serum IgG	3.3 g/dl				0.7–1.5 g/dl
ASMA	Positive				
Brucella SAT	Negative				
Serum Protein Electrophoresis	Polyclonal hypergammaglobulinemia				
Blood Film Morphology	Anisocytosis, red blood cell (RBC) agglutination				
Fibro-scan	19 kPa				<7.0 kPa (normal); >14 kPa = cirrhosis
ABG (Lactate)	19 mmol/L				0.5–2.2 mmol/L

Differentials of malignancy - lymphoma and plasma cell dyscrasia, autoimmune disease and infection, including brucellosis, histoplasmosis and tuberculosis (TB) - were considered. Serum protein electrophoresis showed polyclonal hypergammaglobulinemia and no M spike. Bone marrow biopsy was done and was found to be hypercellular for age with focal clusters of CD3- and CD20-positive cells. The possibility of plasma cell dyscrasia was ruled out. Brucella SAT (standard agglutination test) was sent and found to be negative. Anti-nuclear antibody (ANA) was negative. Complement 3 and 4 (C3 and C4) levels were found to be low. Cervical lymph node biopsy was done and sent for histopathological examination.

The patient was then worked up for chronic liver disease (CLD). Fibro scan was done, which showed a stiffness score of 19 kPa. Upper gastrointestinal endoscopy was done to rule out esophageal varices and was suggestive of low-grade esophagitis. Autoimmune hepatitis panel, inclusive of anti-smooth muscle antibody (ASMA), anti-LKM (liver-kidney microsomal antibody test) and immunoglobulin G (IgG), was sent, out of which ASMA was positive and IgG (3.3 g/dl) levels were raised. Liver biopsy was done, which showed moderate chronic inflammatory infiltrate in the portal tract consisting of lymphocytes and occasional plasma cells.

**Figure 1:**
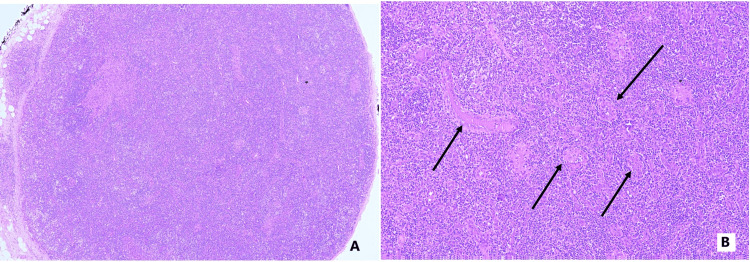
Lymph node showing AITL: effaced architecture, high endothelial venules and polymorphous cell infiltrate are observed A: Low-power photomicrograph showing a lymph node with partly effaced architecture. There is extracapsular extension of the atypical lymphocytes in the adjacent fibroadipose tissue (top left) (H&E; 4x). B: High-power image of lymph node. Black arrows show the prominence of high endothelial venules along with a polymorphous population of cells (H&E; 20x). AITL: angioimmunoblastic T-cell lymphoma

Lymph node (LN) biopsy showed partly effaced architecture with peripherally placed focal-preserved lymphoid follicles, many atretic follicles, and focal loss of capsule and extracapsular lymphoid cells. There was prominence of high endothelial venules. Many plasma cells and a few eosinophils were noted, along with a polymorphous population of lymphoid cells comprising mature lymphocytes, centrocytes, centroblasts and immunoblasts (Figure 1). On immunohistochemistry, CD3, CD20 and CD 79a highlighted heterogeneous lymphoid and T-lymphoid population, with presence of CD8-positive T lymphocytes as well. CD10-positive T cells were focally seen outside the rim of the follicles. CD23- and CD21-positive dendritic meshwork was noted in the germinal center as well as focally around the high endothelial venules. The findings were suggestive of angioimmunoblastic T cell lymphoma.

Initially. the patient was admitted to the Intensive care unit (ICU) on oxygen support, was managed for anemia in failure and was transfused 1 unit packed red blood cells (PRBC). He was started on oral Prednisolone for AIHA. The patient improved clinically and was then transferred to the general ward. 

The patient was planned for two doses of cyclophosphamide 500 mg, but on the next day of receiving the first dose, the patient developed shortness of breath, had hypotension, tachycardia and petechial rash over the abdomen. ABG showed lactic acidosis (lactate: 19mmol/L). The patient was shifted back to the ICU, started on vasopressors, and given IV fluids and broad-spectrum antibiotics. He was intubated in view of a drop in sensorium. Metabolic acidosis worsened further. The patient had a cardiac arrest and could not be revived in spite of cardiopulmonary resuscitation.

## Discussion

The understanding of AITL, which is a rare type of non-Hodgkin lymphoma (NHL) and a subtype of PTCL has evolved over the years from initially being described as non-neoplastic hyperimmune reaction, where it was referred to by various names such as immunodysplastic disease, immunoblastic lymphadenopathy (IBL) and angioimmunoblastic lymphadenopathy with dysproteinaemia (AILD), to being classified under lymphomas by WHO due to its malignant nature being derived from T-follicular helper (Tfh) cells [4].

It presents with a myriad of clinical features such as B symptoms, lymphadenopathy, hepatosplenomegaly and lab findings such as hematologic abnormalities like anemia, lymphopenia, thrombocytopenia or eosinophilia, elevated lactate dehydrogenase, and polyclonal hypergammaglobulinemia [3]. It may mimic an immune activator, exhibiting elevated sedimentation rates and yielding positive results on autoimmune tests such as Coombs test, rheumatoid factor and anti-smooth muscle antibodies. Additionally, it may manifest through circulating immune complexes or cold agglutinins [5].

The regulation of Tfh cells plays a crucial role in maintaining immune balance and preventing autoimmune disorders. Tfh cells are instrumental in orchestrating germinal center reactions, where B-cells undergo somatic hypermutation and affinity maturation to produce high-affinity antibodies. However, dysregulation of Tfh cells can disrupt this process, leading to aberrant germinal center reactions and potentially contributing to the development of autoimmune and inflammatory conditions [5,6].

Our patient's AITL likely contributed to several concurrent autoimmune phenomena including mixed AIHA, positive Anti smooth muscle antibody, elevated IgG levels and hypocomplementemia. Various case reports and observational studies have described AIHA in AITL, mostly being warm AIHA [5,7-9]. Castillo et al. reported a case of AITL wherein patient had mixed AIHA and false-positive HIV enzyme-linked immunosorbent assay (ELISA) [10]. Another case report by Win et al. showed an association of mixed AIHA with splenic angioimmunoblastic NHL [2]. Crickx et al. conducted a comparative retrospective study between AITL with autoimmune cytopenia (AIC) and AITL without AIC, which suggested that AITL group with AIC had more advanced disease and immune activation with more percentage of patients with autoimmune hemolysis, cold agglutinin disease, immune thrombocytopenic purpura and anti-smooth muscle antibodies [11].

Mixed AIHA is a rare entity accounting for 6.5%-8.3% of the cases of AIHA and poses a therapeutic challenge. Treatment of mixed AIHA with AITL is complicated and involves the management of both the underlying lymphoma as well as the autoimmune process. Corticosteroids, for example, prednisone, are generally the first-line agent for AIHA to control immune-mediated destruction of red cells. Because the condition is associated with AITL, however, immunosuppressive or cytotoxins like rituximab, cyclophosphamide, or cyclosporine can be used to gain control over both the hemolysis and the lymphoma. If the AIHA is steroid-refractory, rituximab is effective, especially for control of the B-cell component, which is typically responsible for autoantibody production. Treatment of the underlying AITL with the appropriate chemotherapy regimens, e.g., CHOP chemotherapy (cyclophosphamide, doxorubicin, vincristine, and prednisone), must be undertaken since remission from the lymphoma can resolve the hemolytic anemia. Supportive therapy, such as transfusions and folate supplementation, is also necessary, and treatment is monitored carefully because of the danger of treatment-induced immunosuppression and infection [12]. Our patient was treated with glucocorticoids with a good response initially. The patient was planned for chemotherapy. On receiving the first dose of cyclophosphamide, the patient's condition deteriorated and he eventually died.

## Conclusions

AITL and mixed AIHA are rare and diagnostically challenging conditions that often present with overlapping clinical features. Early recognition of immune-mediated cytopenias in the context of lymphoproliferative disorders is crucial. Effective management relies on a combination of immunosuppressive therapy (e.g., corticosteroids, rituximab) and definitive lymphoma-directed chemotherapy (e.g., CHOP or CHOEP (cyclophosphamide doxorubicin vincristine etoposide prednisolone) regimens). Multidisciplinary care, close monitoring for treatment response and complications, and consideration of targeted therapies when appropriate are key to improving outcomes in these complex cases.
